# Hepatocellular carcinoma: thyroid hormone promotes tumorigenicity through inducing cancer stem-like cell self-renewal

**DOI:** 10.1038/srep25183

**Published:** 2016-05-12

**Authors:** Tao Wang, Lei Xia, Sicong Ma, Xingxing Qi, Qigen Li, Yun Xia, Xiaoyin Tang, Dan Cui, Zhi Wang, Jiachang Chi, Ping Li, Yu-xiong Feng, Qiang Xia, Bo Zhai

**Affiliations:** 1 Department of Interventional Oncology, Renji Hospital, School of Medicine, Shanghai Jiaotong University, Shanghai, PRC, 200127; 2 Department of Hepatic surgery, Renji Hospital, School of Medicine, Shanghai Jiaotong University, Shanghai, PRC, 200127.

## Abstract

Cancer stem-like cells (CSCs) play a key role in maintaining the aggressiveness of hepatocellular carcinoma (HCC), but the cell-biological regulation of CSCs is unclear. In the study, we report that thyroid hormone (TH) promotes cell self-renewal in HCC cells. TH also increases the percentage of CD90 + HCC cells and promotes drug resistance of HCC cells. By analyzing primary human HCC samples, we found that TRα transcript level is significantly elevated in primary liver cancer and portal vein metastatic tumor, compared to that of adjacent normal liver tissue. Knocking down TRα not only inhibits HCC self-renewal *in vitro* but also suppresses HCC tumor growth *in vivo*. Interestingly, treatment of TH leads to activation of NF-κB, which is required for the function of TH on inducing HCC cell self-renewal. We also found TRα and p65 cooperatively drive the expression of BMI1 by co-binding to the promoter region of *BMI1* gene. In summary, our study uncovers a novel function of TH signaling in regulating the CSCs of HCC, and these findings might be useful for developing novel therapies by targeting TH function in HCC cells.

Hepatocellular carcinoma (HCC) is currently the second most common cause of cancer-associated death worldwide^1^. In the past decade, the incidence of HCC has been increasing, with nearly 800,000 new cases reported every year. HCC is notoriously aggressive, as a diversity of malignant features, including vast capacity of tumor expansion, intrinsic multi-drug resistance, and extraordinary tumor seeding and metastatic potential, usually appear concomitantly in HCC cells^2^. The involvement of cancer stem-like cells (CSCs) has been proposed to play a key role in maintaining the aggressiveness of HCC, since it is known that cancer stem-like cells contribute to multiple features above in many types of cancer^3^. However, to date it remains largely unknown how CSCs of the liver cancer are maintained and regulated, albeit some cell-surface markers to enrich CSCs have been discovered. It has been shown that CD90 positive (CD90+) and EpCAM positive cancer cells function as cancer stem-like cells in liver cancers^4^,^5^.

Hormone and nuclear receptor (NR) signaling pathways are essential in regulating gene expression^6^. NRs are a group of transcriptional factors. Once activated by their cognate ligands, NRs activate the transcription of the target genes, which in turn modulate various cell-biological and developmental processes. Recently studies demonstrated that many NRs are involved in regulating stem cell self-renewal and proliferation in various types of tissue during development^7^. Although the roles of NRs in many types of cancer development are established, it is not clear whether NRs are important in regulating CSCs activity. Latest studies showed that estrogen and estrogen receptor ERα expand a pool of functional breast CSCs cells through a paracrine FGC/FGFR/Tbx3 signaling pathway^8^. Testicular receptor (TR4) promotes prostate cancer initiation in peroxisome proliferator-activated receptor gamma deleted prostate cells^9^. These all suggest that NRs could affect cancer progression via regulating CSCs.

The function of TH in cancer development has been known long ago^10^. Recent studies indicated that subclinical hyperthyroidism might increase the risk of certain solid tumors^11^, while spontaneous hypothyroidism may delay onset or reduce aggressiveness of cancers. However, how TH plays a role in liver cancer remains unknown. In this study, we found that TH significantly increased cell self-renewal in HCC cells. TH also increases the number of CD90 + HCC cells and promoted drug resistance in HCC cells. The function of TH was clearly through its receptor alpha (TRα). Loss-of-function experiments revealed that decreasing TRα expression significantly suppressed both *in vitro* cell self-renewal and *in vivo* tumor growth of HCC cells. Interestingly, by analyzing primary human HCC samples, we found that TRα transcript level was significantly elevated in primary liver cancer and portal vein metastatic tumor, compared to that in adjacent normal liver tissue. Furthermore, we found that the function of TH signaling co-operatives with NF-κB in HCC cells. TRα interacts with NF-κB subunit p65 and co-occupies the promoter region of oncogene *BMI1* in TH-treated HCC cells. In summary, our study demonstrated a critical role of TH signaling in self-renewal of liver CSCs.

## Materials and Methods

### HCC cDNA samples and cell lines

The HCC normal/primary tumor (PT)/portal vein tumor thrombosis (PVTT) cDNA samples were obtained from Renji Hospital, Shanghai Jiao Tong University. Informed consent was obtained from all patients. Tissue biopsy was approved by the Institutional Review Board of the Renji Hospital, Shanghai Jiao Tong University. All the experiments using human tissue samples were carried out in accordance with the guidelines approved by the Institutional Review Board of the Renji Hospital, Shanghai Jiao Tong University. The CSQT-2 cell was prepared from an *in vitro* culture of a PVTT-1 xenograft that was established previously^12^, and were cultured in DMEM, supplemented with 10% fetal bovine serum, 10 units/ml penicillin, and 10 units/ml streptomycin, at 37 °C in a humidified atmosphere containing 5% CO_2_.

### Reagents and plasmids

All 24 small molecules used in the screen (supplemental table 1) were purchased from Sigma. Anti-human CD90, CD133, and EpCAM antibodies for flowcytometry analysis were from BD Biosciences. Methylcellulose was from R&D Systems. pLKO-shTHRA and pLKO-shTHRB constructs were from Sigma. shRNA sequences: *THRA* shRNA-1 5′-GTCAGGGTATATCCCTAGTTA; *THRA* shRNA-2 5′ CAAACACAACATTCCGCACTT; *THRB* shRNA-1: 5′ GCCTGTGTTGAGAGAATAGAA; *THRB* shRNA-2: 5′ CCACTTGGACTAGCTCAATAT; p65 (*RELA*) shRNA GCCTTAATAGTAGGGTAAGTT;

### Immunoprecipitation

Immunoprecipitation was conducted as previously described^14^. Briefly, whole cell lysates were prepared from 5 x 107 CSQT-2 cells treated by T4 for 96 hrs using RIPA lysis buffer with protease inhibitor cocktail. Nuclear fraction of protein from CSQT-2 Cell lysates was harvested. The lysates were pre-cleared by incubating with protein A-Sepharose for 1 h at 4°C and centrifugation. The supernatant was immunoprecipitated with 1 μg rabbit IgG or anti-p65 antibody overnight at 4°C. Immune complexes were collected by incubation with protein A-Sepharose for 4 hrs at 4°C and washed for 5 times at 4°C with lysis buffer. The immune complexes adsorbed to the beads were centrifuged and the supernatant was removed. 50 μL of 1x loading buffer was added to the samples and boiled at 95°C for 5 minutes. Proteins were resolved by SDS-PAGE and immunoblotted by antibodies indicated in figures.

### *In vitro* colony formation assay

1000 liver cancer cells were seeded in 3.5 cm dish with 2 ml of culture media, and cultured for up to 5 days. Cell-colony forming was measured by crystal violet staining at day 5^15^. The data was analyzed by the ImageJ software.

### *In vitro* self-renewal assay

1,000 liver cancer cells were seeded with DMEM containing 20% methylcellulose (Stemcell Technologies) in ultra-low attachment plate (Corning) for a period of 7 days^13^. Chemicals were added into the culture media at 10 μM the same day when cells were seeded. The number and size of colonies formed were quantified at day 7. The data was analyzed using the software Image-J.

### *In vivo* tumorigenicity assay

Male NOD/SCID mice were housed under standard conditions. The animal protocols were done in agreement with Renji Hospital Guide for the Care and Use of Laboratory Animals and approved by Animal Care and Use Committee, Renji Hospital. Six-week-old male NOD/SCID mice were subcutaneously injected at two flanks and/or the back with 10^3^, 10^4^, 10^5^, or 10^6^ liver cancer cells per sites. The resulting tumors were measured with calipers every 7 days, and tumor volume (mm^3^) was calculated using the standard formula: length × width × height × 0.5236. Tumors were harvested from ether-anesthetized mice. All the animal protocols were reviewed and approved by the Experimental Animal Research Committee of the Renji Hospital, Shanghai Jiao Tong University. Stem cell frequency in the tumor was analyzed by Extreme Limiting Dilution Analysis (http://bioinf.wehi.edu.au/software/elda/).

### Statistical analysis

All data were expressed as mean ± SEM. For comparison of two different groups, Student’s t test was used. Differences between groups were considered significant at p < 0.05.

## Results

### Identification of thyroid hormone as a potent factor promoting cancer stem-like cell phenotypes in HCC

**Figure 1:**
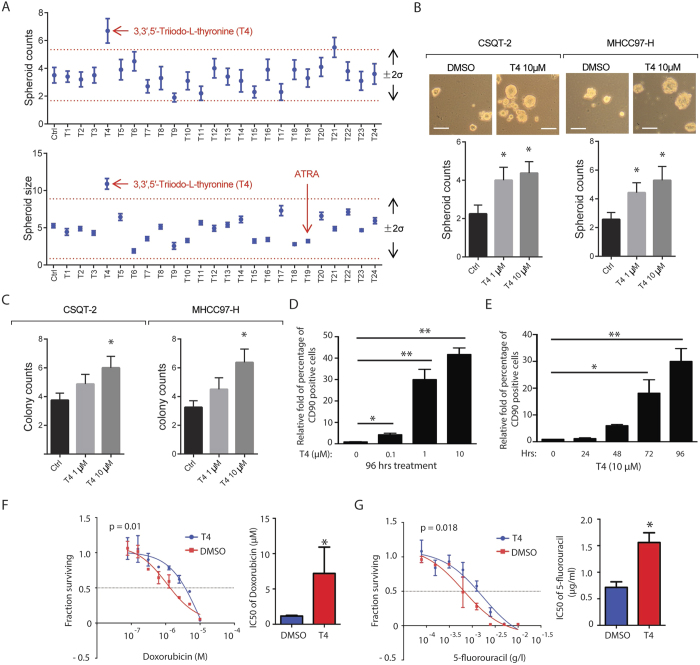
TH stimulates self-renewal and enhances drug-resistance in HCC cells. (**A**) CSQT-2 cells were seeded in 96-well ultra-low attachment plates with DSMO or 24 different hormone-related chemicals (see methods and supplementary table 1 for details) for 7 days. The spheroid-genesis in each condition was measured by spheroid counts per bright-field area as well as the size of spheroids. The range of two standard deviations was indicated. The number of spheroids in 10 fields per condition was counted, and the size of 20 spheroids in each condition was measured. (**B**) CSQT-2 and MHCC97-H cells were seeded in 6-well ultra-low attachment plates, and treated with DMSO and 10 μM T4 (3, 3′, 5′-Triiodo-L-thyronine) for 7 days. Representative images and quantification of spheroid counts were shown. (**C**) CSQT-2 and MHCC97-H cells were seeded in 6-well cell culture plates (500 cell per well), and treated with DMSO or T4 for 5 days. The number of colonies formed was measured at day 5. (**D**) Flow cytometry analyses of CSQT-2 cells treated with indicated concentrations of T4 for 96 hours, and the fold change of CD90+ cells were plotted (The percentage of CD90+ cells in DMSO-treated condition was set to be “1”). (**E**) Flow cytometry analyses of CSQT-2 cells treated with 10 μM T4 for indicated period of time, and the fold change of CD90+ cells were plotted (The percentage of CD90+ cells in DMSO-treated condition was set to be “1”). (**F**,**G**) CSQT-2 cells were treated with a series doses of Doxorubicin or 5-Fluorouracil in the presence or absence of 1 μM T4 for 3 days. Cell survival was measured using the CellTiter-Glo assay (Promega). IC50 was simulated using Prism 5. Error bars represent mean ± SEM from three biological replicates, unless otherwise indicated. (*p < 0.05, **p < 0.01, ***p < 0.001).

To identify hormone signaling that affects cancer stem-like cells of HCC (HCC-CSCs), we conducted a tumor spheroid-based screening on CSQT-2 cell. CSQT-2 is a highly aggressive HCC cell line originated from a specimen of portal vein tumor thrombus (PVTT)^12^. We have reported that CSQT-2 manifests many features of advanced HCC, including potent self-renewal capacity, multi-drug resistance, and vasculature metastatic potential^12^. In a non-adherent stem-cell culture condition, CSQT-2 cells were treated with a set of 24 agonists or antagonists of nuclear receptors, and the number and size of tumor spheroids in each condition were measured at day 7 (Supplemental Table 1). Most of the compounds lead to a fold-change within two standard deviations of the changes across all treatments (Fig. 1A). Interestingly, only one molecule, T4 (Thyroxine), a ligand of the thyroid hormone receptor signaling, caused an increase over two standard deviations for both number and size of spheroids formed (Fig. 1A), strongly indicating that T4 was a potent factor promoting the self-renewal of HCC-CSCs. In contrast, ATRA, a known factor that differentiates HCC-CSCs, was able to suppress the sphere formation, indicated by decreased size of spheroids formed (Fig. 1A).

We then validated the function of T4 in another HCC cell line, MHCC97-H, and revealed that treatment of T4 promoted the sphere formation in both CSQT-2 and MHCC97-H cells (Fig. 1B). In addition, we also examined the effect of T4 on the self-renewal of HCC-CSC using a 2D colony-forming assay. As expected, T4 led to a two-fold increase of the number of colonies in both HCC cells (Fig. 1C). Consistently, treatment of T4 dramatically induced the percentage of CD90 + HCC cells in a dose and time dependent manner (Figs 1D,E and S1). As controls, we tested the function of three different agonists targeting other NR receptors: peroxisome proliferator-activated receptor gamma (PPARγ), farnesoid X receptor (FXR) and liver X receptor (LXR), on induction of CD90+ cells. These three receptors are highly expressed in liver cells and closely regulate the function in both normal and tumor liver cells. However, none of these agonists increased the percentage of CD90+ CSQT-2 cells (Fig. S1). Since heightened drug resistance is another hallmark of cancer stem-like cells^16^, we next investigated whether TH increases the drug resistance of HCC cells. By use of doxorubicin and 5-fluorouracil, two common chemotherapeutic agents in treating HCC, we found that presence of T4 significantly increases the drug resistance of CSQT-2 cells, elevated by ~8 fold and ~2 fold for Doxorubicin and 5-fluorouracil respectively as determined by IC50 (Fig. 1F,G).

### TH signaling in HCC is through nuclear receptor TRα

**Figure 2:**
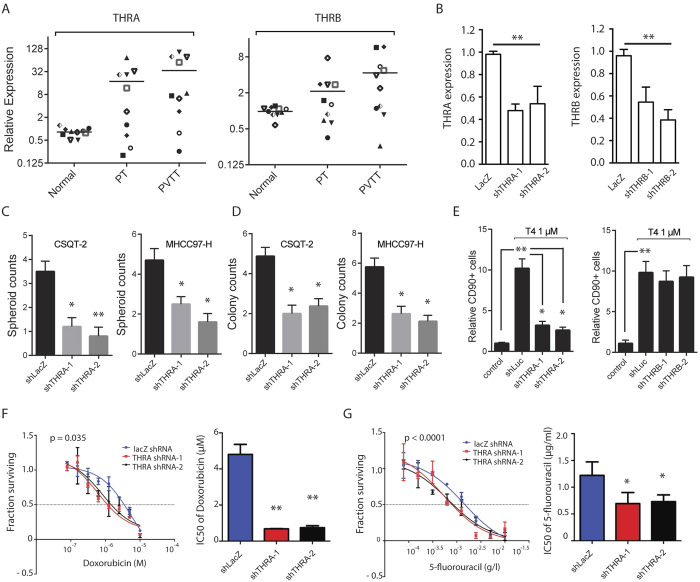
TH signaling in HCC is through receptor TRα. (**A**) Real-time qPCR to quantify expression of *THRA* and *THRB* in HCC patient samples. PT, primary tumor; PVTT, portal vein tumor thrombus. Each dot represents one patient sample. (**B**) Lentivirus encoding shRNA targeting LacZ (control), TRα (*THRA*), or TRβ (*THRB*) was transduced into CSQT-2 cells, and the knockdown efficiency for *THRA* and *THRB* was measured by qPCR. (**C**) Flow cytometry analysis measuring CD90 + CSQT-2 cells were performed 72 hours after shRNA transduction in (**B**). (**D**) Lentivirus encoding shRNA targeting LacZ (control) or TRα (*THRA*) was transduced into CSQT-2 and MHCC97-H cells, and cells were seeded in ultra-low attachment plates in the presence of 1 μM T4 for 7 days. Spheroid-genesis was measured by spheroid counts at day 7. (**E**) 500 Cells from (**D**) were seeded in cell culture plate for 5 days, and number of colonies formed was counted. (**F**) CSQT-2 cells from (**B**) were treated with a series doses of Doxorubicin or 5-Fluorouracil in the presence of 1 μM T4 for 3 days. Cell survival was measured using the CellTiter-Glo assay (Promega). IC50 was simulated using Prism 5. Error bars represent mean ± SEM. from three biological replicates. (*p < 0.05, **p < 0.01, ***p < 0.001).

TH transduces signaling events through interacting with its cognate nuclear receptors. There are two types of TH receptors: TRα and TRβ, encoded by *THRA* and *THRB* respectively in mammals^17^. While TRβ is highly expressed in hepatocytes and mediates thyroid hormone-regulated lipogenesis, the function of TRα in liver is largely unclear^18^. We next decided to test the expression pattern of TH receptors in different tissue samples from HCC patients’ liver biopsy samples, including paired normal liver, primary tumor and PVTT of HCC tissue samples. Our qPCR data showed that although TRα expression was low in normal liver tissues, its expression was much higher in primary tumors. Most importantly, the highest TRα expression, ~32-fold increase compared to that in normal tissue, was detected in PVTT tissues (Fig. 2A). TRβ expression is also induced in primary tumors and PVTT tissues, but to a much less extent compared to that of TRα expression (Fig. 2A). We further confirmed the cellular function of TRα in TH-mediated CSCs self-renewal. Knocking down TRα reduced the number of tumor spheres counts in CSQT-2 cells and MHCC97-H cells by 2 to 3-fold compared to that in control cells with T4 (Fig. 2B,C). Likewise, TRα knockdown led to a significant reduction in the colony forming capabilities of CSQT-2 cells and MHCC97-H cells (Fig. 2D). In contrast, we did not observe any significant changes in sphere formation and colony formation by knocking down TRβ (data not shown). Knocking down TRα but not TRβ also reduce the percentage of CD90 + CSQT-2 cells induced by T4, which further confirms the function of TRα in regulating HCC cell self-renewal (Figs 2E and S2). As expected, knocking down TRα significantly increased the sensitive of HCC cells to treatment of Doxorubicin and 5-fluorouracil, which suggests TRα is essential for TH-promoted drug resistance in HCC cells (Fig. 2F,G).

### **TRα** is essential for tumor initiation and growth *in vivo*

**Figure 3:**
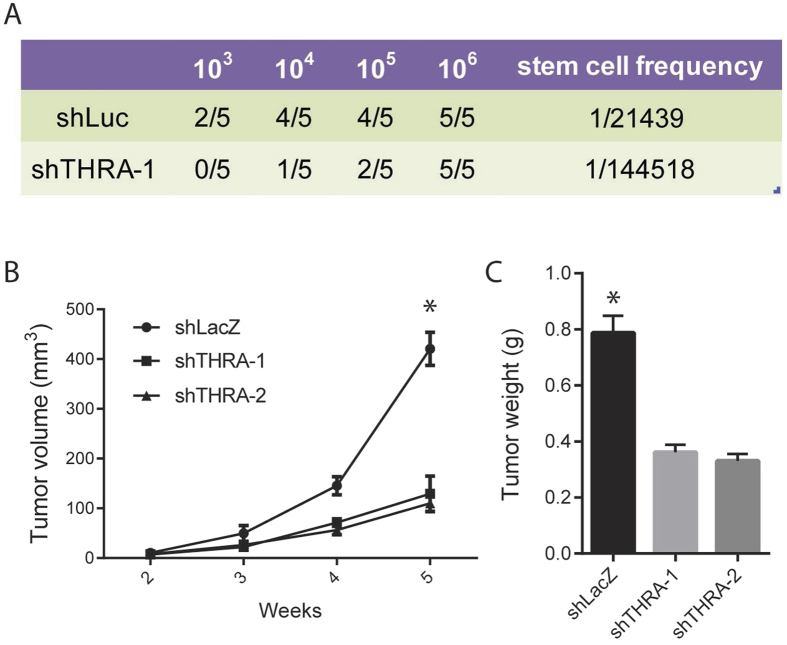
TRα is essential for tumor initiation and growth *in vivo*. (**A**) CSQT-2 cells were transduced by lentivirus encoding LacZ (control) or TRα (*THRA*) shRNAs, and transplanted into NOD/SCID mice at day 3 post-transduction. The number of cells injected was indicated, and the percentages of animals that formed tumors were quantified. The incidence of stem-like cells in the overall population was estimated. For the group of mice injected with 10^6^ cells, the tumor volume (**B**) and tumor weight (**C**) were measured. (*p < 0.05, **p < 0.01, ***p < 0.001).

We next determined whether TRα signaling is essential for HCC tumor growth *in vivo*. We applied the serial dilution injection assay, the golden standard in stem cell biology, to test if TRα signaling affects the percentage of tumor-initiating cells in HCC. Upon TRα knock-down, the abundance of tumor-initiating cells of CSQT-2 cells were only 1/5 compared to the control cells (Fig. 3A). Remarkably, while both cells could initiate tumors when 10^6^ cells were implanted, the control cells initiated tumors much more rapidly than the cells with TRα knockdown, as gauged by tumor volume along time (Fig. 3B). Consequently, mice implanted with TRα knockdown HCC cells had a marked decrease in tumor burden compared to that in control mice as determined by tumor size and weight (Figs 3C and S3). Taken together, our results suggest TH signaling mediated by TRα receptor is important for HCC tumor growth *in vivo*.

### NF-κB cooperates with TRα in regulating gene expression in HCC cells

**Figure 4:**
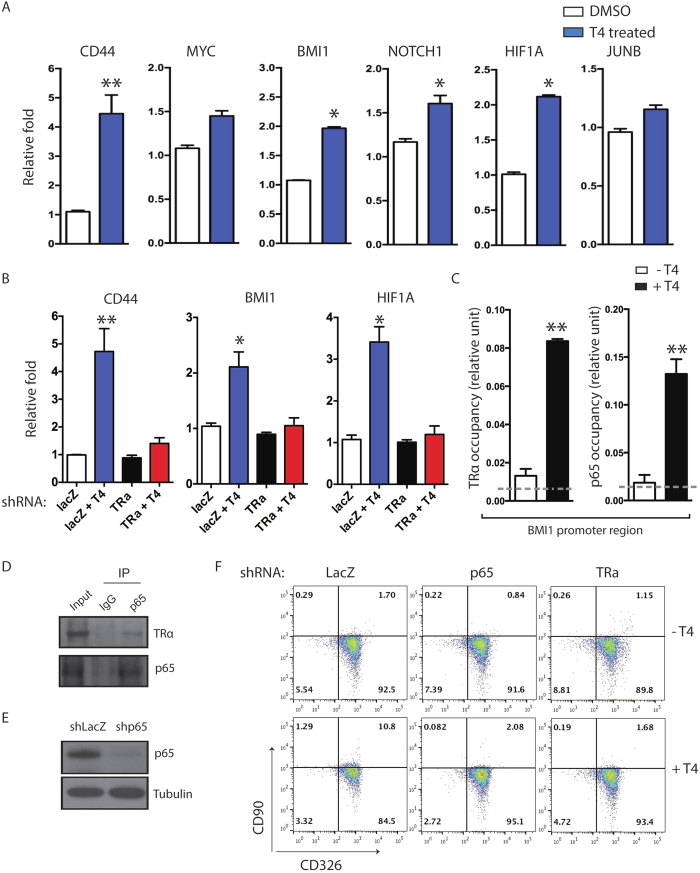
TRα cooperates with NF-κB to regulate stem cell genes expression in HCC cells. (**A**) CSQT-2 cells were treated with DMSO or 1 μM T4, and the expression of CD44, MYC, BMI1, NOTCH1, HIF1A, and JUNB was measured by qPCR. (**B**) Lentivirus encoding shRNAs targeting LacZ (control), or TRα (*THRA*) was transduced into CSQT-2 cells and treated with DMSO or 1 μM T4, and the expression of *CD44*, *BMI1*, and *HIF1A* was measured by qPCR. (**C**) Quantitative ChIP analysis of TRα and p65 occupancy at *BMI1* promoter region in CSQT2 cells following the indicated treatments. Units are arbitrary; signals using rabbit IgG are represented by grey dot lines across the plots. (**D**) Co-immunoprecipitation (IP) measuring interaction between TRα and NF-κB subunit p65 in CSQT2 cells after treated with T4 for 72 hrs. (**E**) Western blot showing the knockdown effect of p65 in CSQT-2 cells transduced with shp65 hairpins. (**F**) Lentivirus encoding shRNA targeting LacZ (control), p65, or TRα was transduced into CSQT-2 cells treated with DMSO or T4. Flow cytometry analyses measuring CD90 and CD326 were performed at 72 hours after transduction. (*p < 0.05, **p < 0.01, ***p < 0.001).

We next sought to understand the molecular mechanism by which TH signaling promotes HCC self-renewal. Although the exact mechanism of cancer stem-like cell self-renewal is not clear, a set of essential markers or regulators are known, including *CD44*, *BMI1, MYC, NOTCH1, HIF1A*, and *JUNB*^4^,^19^,^20^. Our quantitative PCR analyses showed that T4 significantly induces expression of some of these stem cells genes, such as *CD44*, *BMI1, NOTCH1* and *HIF1A,* in CSQT-2 cells (Fig. 4A). Markedly, knocking down TRα impaired the expression of *CD44*, *BMI1* and *HIF1A* (Fig. 4B), suggesting that TRα was involved in the regulation of these gene expression in HCC cells.

Previous studies have demonstrated that NF-κB is usually hyper-activated in HCC and is highly associated with cancer progression^21^. We hypothesized that NF-κB might cooperate with TRα in regulating gene expression in HCC cells. Previous studies have showed that overexpression of *BMI1* in tumor-initiating cells is essential for cancer cell chemo-resistance^22^,^23^,^24^. BMI1 cooperates with other oncogenic signaling pathways to promote hepatic carcinogenesis. Since BMI1 expression is up-regulated by T4 and down-regulated by TRα knockdown, we decided to specifically investigate whether TRα cooperates with NF-κB at *BMI1* genomic locus. Our motif enrichment analysis identified that there were multiple canonical half-sites of thyroid hormone responsive elements (TREs, “AGGTCA”) and a NF-κB binding motif surrounding within 2 kb of *BMI1* transcription start site (TSS). Our ChIP-PCR data demonstrated that while the occupancy of both TRα and NF-κB were low at promoter region of *BMI1* in untreated CSQT-2 cells, the occupancy of both transcription factors were significantly increased in T4-treated CSQT-2 cells (Fig. 4C). In consistency, our immunoprecipitation data suggested that TRα interacted with p65 in T4-treated CSQT-2 cells (Fig. 4D), suggesting TRα might cooperate with NF-κB to regulate gene expression essential for HCC self-renewal. To evaluate the function of NF-κB on TH signaling in CSQT-2 cells, we specifically knocked down p65 gene expression (Fig. 4E). As expected, knocking down p65 in CSQT-2 cells significantly decreases the percentage of CD90+ cells promoted by T4 (Fig. 4F), suggesting that elevated NF-κB signaling is critical in the TH-mediated regulation of HCC CSCs. Taken together, our results highly suggested TRα and NF-κB cooperate with each other to regulate gene expression in TH-treated HCC cells, which in turn enhance the self-renewal of HCC cancer stem-like cells.

## Discussion

In this study, we set out to investigate the function of TH in regulating the CSCs of hepatocellular carcinoma, and revealed that TH can induce HCC cell self-renewal, increase the percentage of CD90 + HCC cells, and promotes drug resistance of HCC cells. These findings were in consistency with recent discoveries that TH plays a part in stem/progenitor cell physiology in other context^25^. We found that this function of TH was, at least in part, via the NF-κB pathway by TH receptor TRα. We also demonstrated that the co-operation between TRα and NF-κB was essential for inducing *BMI1* gene expression in HCC cells (Fig. S4).

HCC is known to be very heterogeneous, yet the mechanism to maintain this intra-tumor complexity is unclear. Our findings suggest a potential role for TH, as a potent extracellular factor, in sustaining or expanding the heterogeneity of HCC cells. Recent studies have shown that increased heterogeneity in liver CSCs results in the heightened chemo-resistance of tumor cells^26^. Indeed, HCC cells appeared to be more resistant to chemotherapy when the TH signaling is active. In consistency with these, our results suggested that TH might specifically promote CD90 + HCC cells and enhance the self-renewal of HCC cells, which subsequently contribute to HCC drug resistance. In the clinical point of view, identifying environmental or genetic factors contributing to cancer heterogeneity is essential for developing efficient therapies to treat liver cancer. Therefore, it will be interesting to investigate the detailed function of TH on liver CSCs heterogeneity on certain liver cancer mouse models in the future.

Although conflicted results have been reported, the notion that TH is involved in liver tumorigenesis is unambiguous^10^. High frequency of somatic point mutations of TRα (65%, 11/17) and TRβ (76%, 13/17) is identified in human HCC^10^,^27^. The function of these mutations has not been verified individually. However, some mutations have been predicted to alter TH receptor DNA binding capability, which in turn will affect their transcriptional activities^10^. Our data suggests a previously unappreciated role of TRα in the self-renewal of HCC both *in vitro* and *in vivo*. Yen *et al.* previously showed that overexpressing TH receptors in HepG2, a non-aggressive cell line in the presence of T3 inhibits cell growth^28^. This could suggest that in the early stage of HCC, TH could have certain anti-proliferation role; however, along with HCC progression, TH could function as an important mediator of aggressiveness and malignancy. This notion could be very well supported by the step-wise increase of expression of thyroid hormone receptor in normal liver, primary tumor, to PVTT. To better understand the role of TH in HCC cells of various genetic background, single-cell based analysis will be required to examine the function of wild type and mutated TH receptors in different tissues, and how they contribute to the disease progression.

Recent studies have revealed the role of NF-κB in CSCs^25^,^29^,^30^. NF-κB has been shown to be activated in ovarian cancer stem cells, where it can inhibit apoptosis, stimulate cell proliferation and tumor growth and resistance to chemotherapy^31^,^32^. In liver CSCs, Cao *et al.* reported that osteopontin promotes a cancer stem-like phenotype in HCC cells through an integrin- NF-κB- HIF1α pathway and up-regulates the expression of HIF1α downstream gene *BMI1*, which is essential for mediating the maintenance of the stem-like phenotypes^33^. In our studies, we found NF-κB was activated in TH-treated HCC cells and contributed to the cancer stem-like phenotypes in HCC cells. We further found that TH receptor TRα interacted with NF-κB subunit p65 and co-occupied the promoter region of *BMI1* in TH-treated HCC cells. Since the cooperation between NF-κB and nuclear receptors including TH receptors is important in regulating hormone-mediated gene expression^34^,^35^, our studies elucidate a key mechanism underlying the self-renewal of HCC induced by TH and potentially useful for developing therapies to treat HCC.

## Additional Information

**How to cite this article**: Wang, T. *et al.* Hepatocellular carcinoma: thyroid hormone promotes tumorigenicity through inducing cancer stem-like cell self-renewal. *Sci. Rep.* **6**, 25183; doi: 10.1038/srep25183 (2016).

## Supplementary Material

Supplementary Information

## References

[b1] Altekruse S. F., McGlynn K. A. & Reichman M. E. Hepatocellular carcinoma incidence, mortality, and survival trends in the United States from 1975 to 2005. Journal of clinical oncology: official journal of the American Society of Clinical Oncology 27, 1485–1491, doi: 10.1200/JCO.2008.20.7753 (2009). 19224838 PMC2668555

[b2] Carr B. I. Hepatocellular carcinoma: current management and future trends. Gastroenterology 127, S218–224 (2004). 15508087 10.1053/j.gastro.2004.09.036

[b3] Yamashita T. & Wang X. W. Cancer stem cells in the development of liver cancer. The Journal of clinical investigation 123, 1911–1918, doi: 10.1172/JCI66024 (2013). 23635789 PMC3635728

[b4] Mani S. A. *et al.* The epithelial-mesenchymal transition generates cells with properties of stem cells. Cell 133, 704–715, doi: 10.1016/j.cell.2008.03.027 (2008). 18485877 PMC2728032

[b5] Yamashita T. *et al.* EpCAM-positive hepatocellular carcinoma cells are tumor-initiating cells with stem/progenitor cell features. Gastroenterology 136, 1012–1024, doi: 10.1053/j.gastro.2008.12.004 (2009). 19150350 PMC2828822

[b6] Aranda A. & Pascual A. Nuclear hormone receptors and gene expression. Physiological reviews 81, 1269–1304 (2001). 11427696 10.1152/physrev.2001.81.3.1269

[b7] Jeong Y. & Mangelsdorf D. J. Nuclear receptor regulation of stemness and stem cell differentiation. Experimental & molecular medicine 41, 525–537, doi: 10.3858/emm.2009.41.8.091 (2009). 19696553 PMC2739892

[b8] Fillmore C. M. *et al.* Estrogen expands breast cancer stem-like cells through paracrine FGF/Tbx3 signaling. Proceedings of the National Academy of Sciences of the United States of America 107, 21737–21742, doi: 10.1073/pnas.1007863107 (2010). 21098263 PMC3003123

[b9] Lin S. J. *et al.* TR4 nuclear receptor enhances prostate cancer initiation via altering the stem cell population and EMT signals in the PPARG-deleted prostate cells. Oncoscience 2, 142–150 (2015). 25859557 10.18632/oncoscience.121 PMC4381707

[b10] Wu S. M., Cheng W. L., Lin C. D. & Lin K. H. Thyroid hormone actions in liver cancer. Cellular and molecular life sciences: CMLS 70, 1915–1936, doi: 10.1007/s00018-012-1146-7 (2013). 22955376 PMC11113324

[b11] Hercbergs A. H., Ashur-Fabian O. & Garfield D. Thyroid hormones and cancer: clinical studies of hypothyroidism in oncology. Current opinion in endocrinology, diabetes, and obesity 17, 432–436, doi: 10.1097/MED.0b013e32833d9710 (2010). 20689420

[b12] Wang T. *et al.* Characterisation of a novel cell line (CSQT-2) with high metastatic activity derived from portal vein tumour thrombus of hepatocellular carcinoma. British journal of cancer 102, 1618–1626, doi: 10.1038/sj.bjc.6605689 (2010). 20461085 PMC2883151

[b13] Feng Y. X. *et al.* Epithelial-to-Mesenchymal Transition Activates PERK-eIF2alpha and Sensitizes Cells to Endoplasmic Reticulum Stress. Cancer discovery 4, 702–715, doi: 10.1158/2159-8290.CD-13-0945 (2014). 24705811

[b14] Lee H. Y. *et al.* PPAR-alpha and glucocorticoid receptor synergize to promote erythroid progenitor self-renewal. Nature 522, 474–477, doi: 10.1038/nature14326 (2015). 25970251 PMC4498266

[b15] Feng Y. X. *et al.* Sorafenib suppresses postsurgical recurrence and metastasis of hepatocellular carcinoma in an orthotopic mouse model. Hepatology 53, 483–492, doi: 10.1002/hep.24075 (2011). 21274870

[b16] Hanahan D. & Weinberg R. A. Hallmarks of cancer: the next generation. Cell 144, 646–674, doi: 10.1016/j.cell.2011.02.013 (2011). 21376230

[b17] Brent G. A. Mechanisms of thyroid hormone action. The Journal of clinical investigation 122, 3035–3043, doi: 10.1172/JCI60047 (2012). 22945636 PMC3433956

[b18] Erion M. D. *et al.* Targeting thyroid hormone receptor-beta agonists to the liver reduces cholesterol and triglycerides and improves the therapeutic index. Proceedings of the National Academy of Sciences of the United States of America 104, 15490–15495, doi: 10.1073/pnas.0702759104 (2007). 17878314 PMC1978486

[b19] Park I. K., Morrison S. J. & Clarke M. F. Bmi1, stem cells, and senescence regulation. The Journal of clinical investigation 113, 175–179, doi: 10.1172/JCI20800 (2004). 14722607 PMC311443

[b20] Lawson D. A. *et al.* Single-cell analysis reveals a stem-cell program in human metastatic breast cancer cells. Nature 526, 131–135, doi: 10.1038/nature15260 (2015). 26416748 PMC4648562

[b21] Luedde T. & Schwabe R. F. NF-kappaB in the liver–linking injury, fibrosis and hepatocellular carcinoma. Nature reviews. Gastroenterology & hepatology 8, 108–118, doi: 10.1038/nrgastro.2010.213 (2011). 21293511 PMC3295539

[b22] Valk-Lingbeek M. E., Bruggeman S. W. & van Lohuizen M. Stem cells and cancer; the polycomb connection. Cell 118, 409–418, doi: 10.1016/j.cell.2004.08.005 (2004). 15315754

[b23] Abdouh M. *et al.* BMI1 sustains human glioblastoma multiforme stem cell renewal. The Journal of neuroscience: the official journal of the Society for Neuroscience 29, 8884–8896, doi: 10.1523/JNEUROSCI.0968-09.2009 (2009). 19605626 PMC6665439

[b24] Molofsky A. V. *et al.* Bmi-1 dependence distinguishes neural stem cell self-renewal from progenitor proliferation. Nature 425, 962–967, doi: 10.1038/nature02060 (2003). 14574365 PMC2614897

[b25] Sirakov M., Skah S., Nadjar J. & Plateroti M. Thyroid hormone’s action on progenitor/stem cell biology: new challenge for a classic hormone? Biochimica et biophysica acta 1830, 3917–3927, doi: 10.1016/j.bbagen.2012.07.014 (2013). 22890105

[b26] Friemel J. *et al.* Intratumor heterogeneity in hepatocellular carcinoma. Clinical cancer research: an official journal of the American Association for Cancer Research 21, 1951–1961, doi: 10.1158/1078-0432.CCR-14-0122 (2015). 25248380

[b27] Chan I. H. & Privalsky M. L. Thyroid hormone receptor mutants implicated in human hepatocellular carcinoma display an altered target gene repertoire. Oncogene 28, 4162–4174, doi: 10.1038/onc.2009.265 (2009). 19749797 PMC2787677

[b28] Yen C. C. *et al.* Mediation of the inhibitory effect of thyroid hormone on proliferation of hepatoma cells by transforming growth factor-beta. Journal of molecular endocrinology 36, 9–21, doi: 10.1677/jme.1.01911 (2006). 16461923

[b29] Yamamoto M. *et al.* NF-kappaB non-cell-autonomously regulates cancer stem cell populations in the basal-like breast cancer subtype. Nature communications 4, 2299, doi: 10.1038/ncomms3299 (2013). 23934482

[b30] Shostak K. & Chariot, A. NF-kappaB, stem cells and breast cancer: the links get stronger. Breast cancer research: BCR 13, 214, doi: 10.1186/bcr2886 (2011). 21867572 PMC3236328

[b31] Chefetz I., Holmberg J. C., Alvero A. B., Visintin I. & Mor G. Inhibition of Aurora-A kinase induces cell cycle arrest in epithelial ovarian cancer stem cells by affecting NFkB pathway. Cell cycle 10, 2206–2214 (2011). 21623171 10.4161/cc.10.13.16348 PMC3154367

[b32] Alvero A. B. *et al.* Molecular phenotyping of human ovarian cancer stem cells unravels the mechanisms for repair and chemoresistance. Cell cycle 8, 158–166 (2009). 19158483 10.4161/cc.8.1.7533 PMC3041590

[b33] Cao L. *et al.* Osteopontin promotes a cancer stem cell-like phenotype in hepatocellular carcinoma cells via an integrin-NF-kappaB-HIF-1alpha pathway. Oncotarget 6, 6627–6640 (2015). 25749383 10.18632/oncotarget.3113 PMC4466639

[b34] De Bosscher K., Vanden Berghe W. & Haegeman G. Cross-talk between nuclear receptors and nuclear factor kappaB. Oncogene 25, 6868–6886, doi: 10.1038/sj.onc.1209935 (2006). 17072333

[b35] Powers C. A., Mathur M., Raaka B. M., Ron D. & Samuels H. H. TLS (translocated-in-liposarcoma) is a high-affinity interactor for steroid, thyroid hormone, and retinoid receptors. Molecular endocrinology 12, 4–18, doi: 10.1210/mend.12.1.0043 (1998). 9440806

