# Supplementary material for: Hepatocellular carcinoma: thyroid hormone promotes tumorigenicity through inducing cancer stem-like cell self-renewal
Source: Sci Rep. 2016 May 12;6:25183. doi: 10.1038/srep25183 (PMC4865859; doi:10.1038/srep25183)
Supplement: Supplementary Information [file srep25183-s1.pdf]

Hepatocellular carcinoma: thyroid hormone promotes tumorigenicity through inducing cancer stem-like cell self-renewal

Tao Wang[1#], Lei Xia[2#], Sicong Ma[1], Xingxing Qi[1], Qigen Li[2], Yun Xia[2], Xiaoyin Tang[1], Dan Cui[1], Zhi Wang[1], Jiachang Chi[1], Ping Li[1], Yu-xiong Feng[1], Qiang Xia[2]*, Bo Zhai[1]*

[1]Department of Interventional Oncology, Renji Hospital, School of Medicine, Shanghai Jiaotong University, Shanghai, PRC, 200127.

[2]Department of Hepatic surgery, Renji Hospital, School of Medicine, Shanghai Jiaotong University, Shanghai, PRC, 200127.

[#] These authors contributed equally to this study.

[*]Corresponding author:

Qiang Xia, Department of Hepatic surgery, Renji Hospital, School of Medicine, Shanghai Jiaotong University. No. 1630, East Road, The New Pudong district, Shanghai, PRC, 200127. Email：xiaqiang@medmail.com.cn, Tel: 86-13661889035

Bo Zhai, Department of Interventional Oncology, Renji Hospital, School of Medicine, Shanghai Jiaotong University. No. 1630, East Road, The New Pudong district, Shanghai, PRC, 200127. Email：zhaiboshi@sina.com, Tel: 86-13918056019

Supplementary Materials and Methods

Extraction of nuclear protein

Nuclear protein was extracted and purified using the NE-PER kit from ThermoFisher (#78833), following the product's manual. The only modification was to extent the nuclear lysis step from forty minutes to one hour and a half on ice, with 10 times of 10-second vortex.

Immunoprecipitation

Immunoprecipitation was conducted as previously described [14]. Briefly, whole cell lysates were prepared from 5 x 10^7 CSQT-2 cells treated by T4 for 96 hrs using RIPA lysis buffer with protease inhibitor cocktail. Nuclear fraction of protein from CSQT-2 Cell lysates was harvested. The lysates were pre-cleared by incubating with protein A-Sepharose for 1 h at 4°C and centrifugation. The supernatant was immunoprecipitated with 1 μg rabbit IgG or anti-p65 antibody overnight at 4°C. Immune complexes were collected by incubation with protein A-Sepharose for 4 hrs at 4°C and washed for 5 times at 4°C with lysis buffer. The immune complexes adsorbed to the beads were centrifuged and the supernatant was removed. 50 μL of 1x loading buffer was added to the samples and boiled at 95°C for 5 minutes. Proteins were resolved by SDS-PAGE and immunoblotted by antibodies indicated in figures.

Western-blot

Cultured cells were washed twice with PBS and lysed in radioimmunoprecipitation assay (RIPA) buffer for 15 min on ice. Cell lysates were clarified by centrifugation at 10 000 *g* for 15 min, and protein concentration was determined by the Bradford Reagent. Lysates were separated on 10% sodium dodecyl sulfate polyacrylamide gel electrophoresis, proteins were then transferred to Immobilon membrane (Millipore, Bedford, MA, USA) and immunoblotted with indicated antibodies. All immunoblots were visualized by enhanced chemiluminescence. (Pierce, Rockford, IL, USA)

Quantitative Real-Time RT-PCR

Total RNA from cells was purified with TRIzol (Invitrogen). cDNA was prepared from 1 μg RNA.

Reaction mixtures (15 µl) contained 2.0 µl of cDNA, 7.5 µl of SYBR green master mix (Applied Biosystems) and appropriate primers. Product was monitored by SYBR green fluorescence. Control reactions lacking RT yielded little to no signal. Relative expression levels were determined from a delta-delta CT method and were normalized to *18S rRNA* expression. Primer sequences are available upon request.

In vitro colony formation assay

1000 liver cancer cells were seeded in 3.5 cm dish with 2 ml of culture media, and cultured for up to 5 days. Cell-colony forming was measured by crystal violet staining at day 5 [15]. The data was analyzed by the ImageJ software.

Chromatin immunoprecipitation

5x107 CSQT2 cells treated with or without T4 were used per IP. Protein-DNA complexes were crosslinked by incubating the cells with Formaldehyde at 1% final concentration. After sonication (12 × 5 sec), a small fraction of the chromatin was uncross-linked by heating the mixture to 67°C for 4 h, and the average size of the DNA fragments (300–400 bp) was determined by gel electrophoresis to verify effective sonication. The rest of the cross-linked chromatin was immunoprecipitated using anti-p65 and anti-TRα specific antibodies overnight at 4°C. The cross-linked protein was next uncoupled from DNA by heating. DNA was purified using Qiaquick gel extraction columns (Qiagen) and resuspended in 50 µL of H2O. Two microliters of immunoprecipitated sample or 2 µL of diluted input DNA were amplified in a 20-µL reaction volume (final) containing 20 mM Tris-HCl (pH 8.4), 50 mM KCl, 2.5 mM MgCl2, 200 µM each dNTP, 0.8 U of HotStart Taq DNA polymerase (Takara), 100 nM FITC, 1 U of SYBR Green, and the sequence-specific primer pair (5 pmol) with temperature cycles of denaturation for 10 sec at 95°C, annealing for 10 sec at 58°C, and extension for 30 sec at 72°C. The primer sequences used for this assay were hBMI1-F 5' - GAGGTAAGCGCCGAACCAAGG -3'; hBMI1-R 5'-GACACTCGCATCCTGGTAACTGG-3'

Supplementary Figure 1

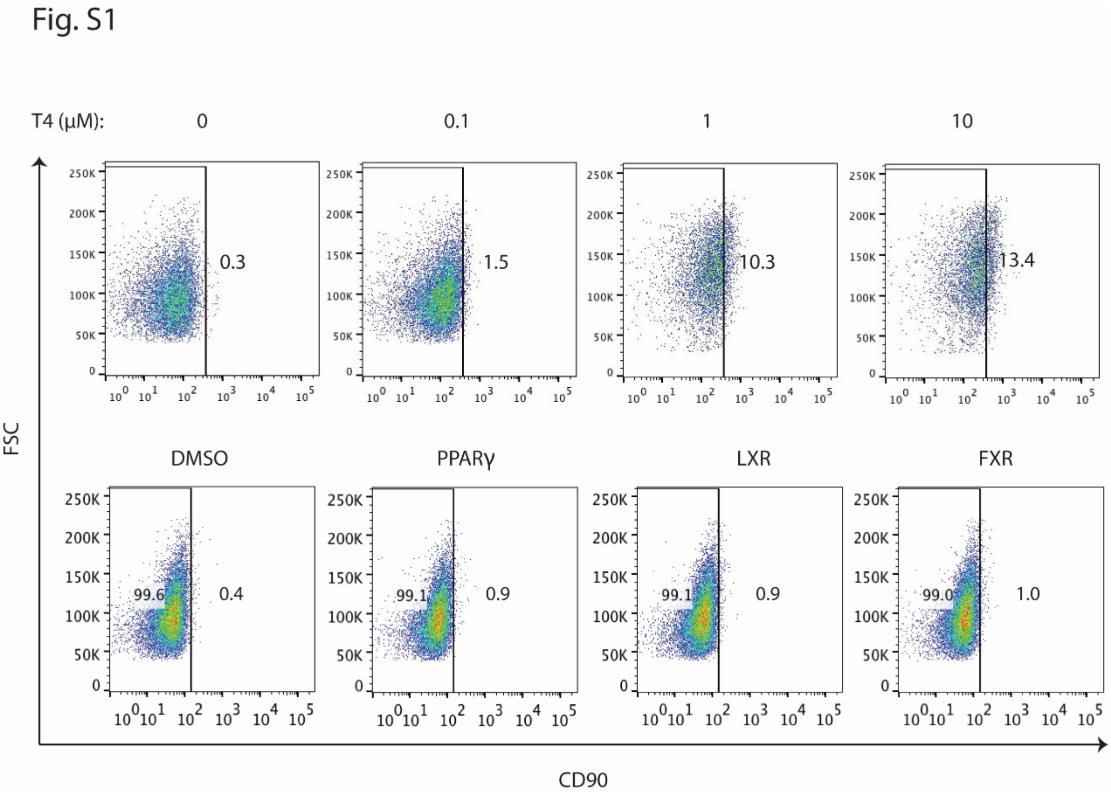

Figure S1. The effects of hormone-related chemicals in the induction of CD90+ HCC cells. Flow cytometry analyses of CSQT-2 cells. CSQT-2 cells were treated by DMSO, a series dose of 3, 3’, 5’-Triiodo-L-thyronine (T4), agonists targeting peroxisome proliferator-activated receptor gamma (PPARγ), farnesoid X receptor (FXR) or liver X receptor (LXR) for 72 hours and stained for CD90. FSC, forward scatter.

Supplementary Figure 2

Fig. S2

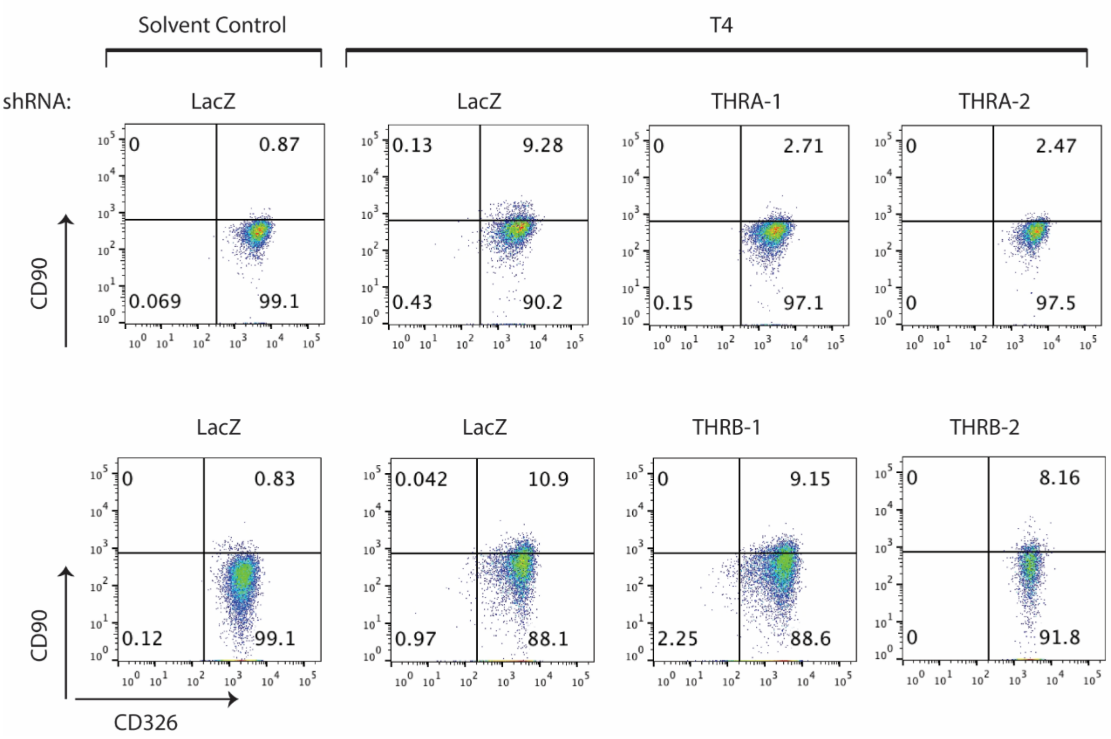

Figure S2. The effects of knocking down THRA and THRB in the induction of CD90+ HCC cells. Lentivirus encoding shRNA targeting LacZ (control), TRα (*THRA*), or TRβ (*THRB*) was transduced into CSQT-2 cells, and the flow cytometry analysis measuring CD90+ CSQT-2 cells were performed 72 hours after shRNA transduction.

Supplementary Figure 3

Fig. S3

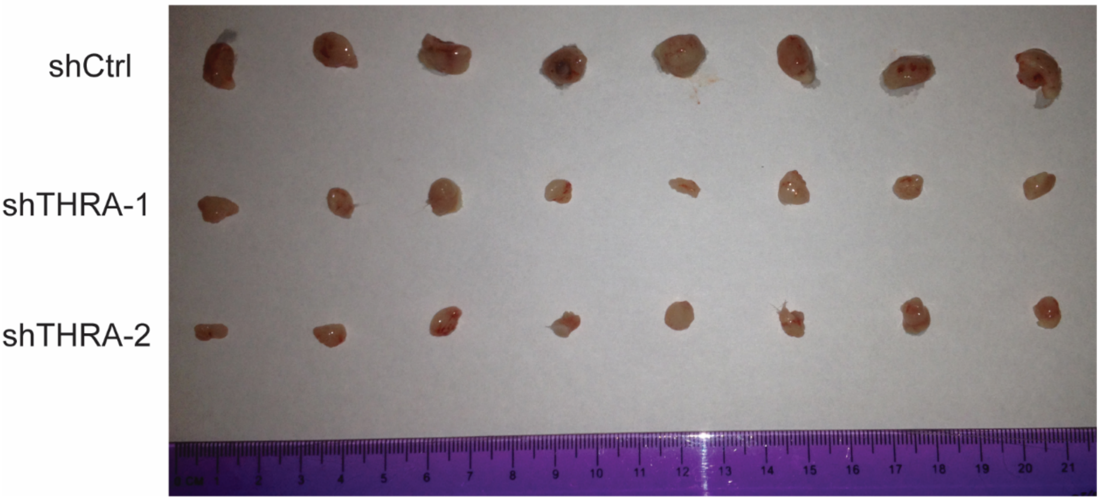

Figure S3. The xenografts formed by CSQT-2 cells with LacZ or TRα knockdown. CSQT-2 cells were transduced by lentivirus encoding LacZ or TRα shRNAs and transplanted into NOD/SCID mice at day 3 post-transduction. Shown were the photomicrographs of tumors formed at 35 days after transplantation.

Figure S4

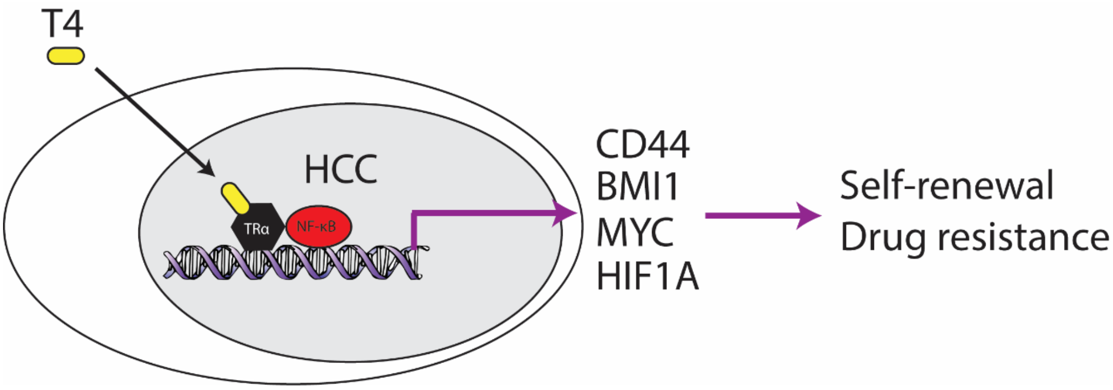

Figure S4 Model of TH signaling in regulating the self-renewal in HCC CSCs.

Supplementary table 1

Order	Compound ID	Information
1	Testosterone	Androgen receptor agonist
2	MDV3100	Androgen receptor inhibitor
3	Doxercalciferol	Vitamin D Receptor Activator
4	Thyroxine	Thyroid hormone receptor agonist
5	Estradiol	estrogen receptor agonist
6	Fulvestrant	estrogen receptor antagonist
7	Rosiglitazone	PPAR gamma agonist
8	GW9662	PPAR gamma agonist
9	Fexaramine	FXR agonist
10	GW3965	LXR agonist
11	Corticosterone	Glucocorticoid receptor agonist
12	Mifepristone	Glucocorticoid and progesterone receptor antagonist
13	Progesterone	Progesterone receptor
14	GW0742	PPARβ/δ agonist
15	GSK0660	potent PPARβ/δ antagonist
16	CP775146	PPAR alpha agonist
17	GW6471	PPAR alpha antagonist
18	UVI 3003	RXR antagonist
19	Retinoic acid	RXR and RAR agonist
20	AM580	RAR alpha agonist
21	BMS 195614	selective RAR alpha antagonist
22	BMS 961	Selective RAR gamma agonist
23	CD 2665	Selective RAR beta/gamma antagonist
24	GSK4716	ERR gamma and ERR beta agonist
